# Not Only Clinical Efficacy in Psychological Treatments: Clinical Psychology Must Promote Cost-Benefit, Cost-Effectiveness, and Cost-Utility Analysis

**DOI:** 10.3389/fpsyg.2016.00563

**Published:** 2016-05-09

**Authors:** Gianluca Castelnuovo, Giada Pietrabissa, Roberto Cattivelli, Gian Mauro Manzoni, Enrico Molinari

**Affiliations:** ^1^Psychology Research Laboratory, Istituto Auxologico Italiano IRCCSVerbania, Italy; ^2^Department of Psychology, Catholic University of MilanMilano, Italy; ^3^Faculty of Psychology, eCampus UniversityNovedrate, Italy

**Keywords:** psychological therapy, cost-effectiveness, evidence-based psychological therapies, evidence-based psychotherapy, evidence based in clinical psychology, mental health burden, psychotherapy

Treating mental disorders is a critical issue for modern societies due to high costs for the different national healthcare systems. Evidence-based psychological therapies and structured psychotherapies have been recommended for common mental health problems, but real provision of them has not yet achieved significant spread and impact (Mukuria et al., 2013).

To improve access to psychological therapies may provide cost-effective solutions, since their positive long-term impact on health has been largely demonstrated (Castelnuovo, 2010a,b; Campbell et al., 2013; Dezetter et al., 2013; Mukuria et al., 2013; Emmelkamp et al., 2014).

However, in many developed countries, such as France or Italy, psychotherapies are not enough covered and promoted by the national healthcare systems and health insurance companies (Dezetter et al., 2013).

Differently, in the UK, to tackle the huge problem of mental illness, a comprehensive programme of psychological therapy has been launched and watched worldwide.

An estimation of its long-term clinical and economic benefits, has, in fact, led to ascertain that “psychological therapy costs nothing” (Layard and Clark, 2014; Clark and Layard, 2015).

In order to mimic the positive experience developed in the UK, other countries have now to demonstrate not only the fundamental role and scientific validity of psychological treatments in both clinical and health settings, but also their significant cost-efficacy.

As noted by Emmelkamp et al. (2014), “There is little doubt from a scientific perspective that psychotherapy according to this definition is effective, highly beneficial and cost-effective for a wide range of mental disorders and health conditions, such as anxiety, stress and trauma-related disorders, depressive and somatoform and pain disorders, personality disorder, substance use disorders and behavioral addictions, eating disorders and a number of childhood disorders. For all these disorders, various variants of CBT have been established in clinical randomized trials” (pp. 66, 67), but research in psychotherapy lacks of cost-benefit analysis of interventions: “even if a psychological treatment could show strong efficacy and/or effectiveness, due to high costs it might never be assimilated in real clinical practice” (p. 67, Emmelkamp et al., 2014).

Unfortunately, most clinical psychologists and psychotherapists are not willing to measure the impact of their clinical practice, even if Beutler (2009) underlined that the gap existing between science and practice could be more imputed to scientists' attitude than to practitioners' intransigence: “scientists were intentionally obscuring many important results because of an unwarranted devotion to a limited number of scientific methods. In fact, I came to believe that they may be using methods and defining psychotherapy and research informed practice in ways that hindered clinicians from being optimally effective” (p. 301, Beutler, 2009).

Considering that psychological therapies should be studied not only in the narrow frame of Empirically Supported Treatments (ESTs) approach, but also using different and integrated research and statistical methods (Beutler, 2009), their cost-effectiveness demonstration remains an unaddressed issue in the psychotherapy field. Indeed, as argued by Lilienfeld et al. (2013) with references to how to promote new evidence-based psychological treatments, “organizational support is often tied to the perceived financial viability of a new treatment (Nelson et al., 2006). Consequently, in order to obtain financial support for learning new approaches and techniques within a clinical context, professionals must first estimate and prove the economic gain of training courses to funding organizations. Treatments that have not demonstrated (or treatments whose cost-effectiveness have not been explored) are therefore less viable options for organizations to support” (p. 895, Lilienfeld et al., 2013).

More clinical studies aimed at providing cost-offset estimations also measuring additional intangible benefits and showing that psychological treatments could be effective, at both clinical and economic levels, are thus necessary (Wunsch et al., 2014).

It is important not to confuse a necessary cost-effective approaches in psychotherapy with dangerous cheap performances provided by the mental health practitioners: “while *cost-effective* treatments can yield savings in healthcare costs, disability claims, and other societal costs, *cost-effective* by no means translates to *cheap* but instead describes treatments that are clinically effective and provided at a cost that is considered reasonable given the benefit they provide, even if the treatments increase direct expenses” (p. 423, Lazar, 2014).

In clinical psychology, standardized, and internationally recognized psychotherapeutic outcome measures have therefore been developed in order to demonstrate patient improvements (Tarescavage and Ben-Porath, 2014), and an ample set of measurements that are useful to evaluate patients outcomes is currently available considering different criteria (administration time and cost, psychometrics and sensitivity to change, etc.). Among these, the Behavior and Symptom Identification Scale-24 (BASIS-24); the Clinical Outcomes in Routine Evaluation Outcome Measure (CORE-OM); the Depression Anxiety Stress Scales (DASS); the Health Survey Short Form-36 (SF-36); the Outcome Questionnaire-45 (OQ-45); the Patient Reported Outcome Measurement Information System (PROMIS); the Symptom Checklist-90-Revised (SCL-90-R); and the Brief Symptom Inventory (BSI) (Tarescavage and Ben-Porath, 2014) are just a few.

However, no agreement has been yet reached over what constitutes relevant ad universal measurements in psychopathology, as requested by the International Consortium for Health Outcomes Measurement (ICHOM) (Porter et al., 2016). Also, clinical psychologists and researchers should strive to develop standardized sets of measures able to estimate savings costs of psychological treatments.

In this regard, commonly used analytic approaches to obtain economic evaluations of health care services are: (1) cost-benefit analysis (focusing on the socially desirable outcome achieved by a particular treatment), (2) cost-effectiveness analysis (taking into account the relationship between monetary costs and measures of treatment outcome, evaluating a possible symptoms reduction or a growing work productivity), (3) cost-utility analysis (with similar features of the cost-effectiveness analysis but using a valuing metric for measuring the treatment impact standardized in terms of quality-adjusted life years—QALY). Considering not only the clinical efficacy of ESTs, but also the number of years of life in which an individual would be expected to be completely free of symptoms or disability is a key point to persuade policy makers and administrators that allocation of resources to psychological interventions would lead to both clinical and economic advantages (Hunsley, 2002).

Taking into account that the future of the health care systems will be the promising stepped care approach for both chronic care pathologies (Davison, 2000; Richards et al., 2003, 2012; Hermens et al., 2014; Castelnuovo et al., 2015a,b; Delgadillo et al., 2015) and mental disorders (Richards, 2012; Watzke et al., 2014; Gidding et al., 2015; Gureje et al., 2015; Haug et al., 2015; Manber et al., 2015; Oladeji et al., 2015; Palmer et al., 2015; Paris, 2015; Salloum et al., 2015; Edelman et al., 2016), clinical psychologists will play a key role in delivering positive outcomes by continuously revising and progressively intensifying the therapeutic process through the reduction of costs.

Despite evidence of the efficacy and effectiveness of the stepped care approach in the clinical field has not been yet reached, positive results have been obtained for preventive treatments aimed at reducing subthreshold depression, which used Internet for individuals who were not able to take part in group interventions (Munoz et al., 1995; Willemse et al., 2004; Van't Veer-Tazelaar et al., 2009; Cuijpers et al., 2010). Instead, another recent study (Van Beljouw et al., 2015) evaluating an outreaching stepped care program on depressive symptoms in older adults failed to demonstrate the utility of the model, while revealing a single (and not stepped) treatment chosen by the participants being sufficient to achieve positive clinical outcomes.

Still, further evidence of the cost-benefit, cost-effectiveness, and cost-utility of both single and stepped care approaches in clinical psychology are needed. Useful indications suggesting how to legitimize clinical psychology in health care system are provided in Table 1.

**Table 1:** **Steps to legitimize clinical psychology in health care system**.

Clinical psychology and psychotherapy should: (1) use ***Research-Supported Psychological Treatments*** as indicated by the *Division 12-Clinica Psychology of the* American Psychological Association (APA) https://www.div12.org/psychological-treatments (Apa Presidential Task Force on Evidence-Based Practice, 2006; Bauer, 2007; Collins et al., 2007; Luebbe et al., 2007; Spring, 2007; Thorn, 2007; Walker and London, 2007; Wampold et al., 2007; Castelnuovo, 2010a; Falzon et al., 2010).
(2) ensure ***clinical efficacy*** through the use of internationally recognized and validated scales such as *Behavior and Symptom Identification Scale-24; Clinical Outcomes in Routine Evaluation Outcome Measure; Depression Anxiety Stress Scales; Health Survey Short Form-36; Outcome Questionnaire-45; Patient Reported Outcome Measurement Information, System; Symptom Checklist-90-Revised and Brief Symptom Inventory* (Tarescavage and Ben-Porath, 2014).
(3) promote ***cost-benefit analysis, cost-effectiveness analysis***, and ***cost-utility analysis*** using internationally recognized tools, as reported by Hunsley (2002), and measure the standardized treatment impact in terms of quality-adjusted life years (QALY*)* (Hunsley, 2002), cost evaluation of healthcare utilization and productivity loss (absenteeism and presenteeism) should be also taken into account, for example using the *Trimbos/iMTA questionnaire for Costs associated with Psychiatric Illness (TiC-P)* (Meuldijk et al., 2015).

Future research in psychology and psychotherapy should, therefore, focus more on cost-effective solutions for the treatment of mental disorders, also considering opportunities provided by new technologies (Castelnuovo et al., 2003, 2014; Cartreine et al., 2010; Castelnuovo and Simpson, 2011; Andrews and Williams, 2014).

## Author contributions

All authors listed, have made substantial, direct, and intellectual contribution to the work, and approved it for publication.

### Conflict of interest statement

The authors declare that the research was conducted in the absence of any commercial or financial relationships that could be construed as a potential conflict of interest.
